# Nasal Cytology Is Useful for Evaluating and Monitoring the Therapeutic Response to Biologics in Chronic Rhinosinusitis with Nasal Polyposis

**DOI:** 10.3390/biomedicines14010077

**Published:** 2025-12-30

**Authors:** Gioia Piatti, Ludovica Battilocchi, Anna Cozzi, Lorenzo Maria Gaini, Mirko Aldè, Lorenzo Pignataro, Sara Torretta

**Affiliations:** 1 Department of Pathophysiology and Transplantation, University of Milan, 20122 Milan, Italy; 2 Department of Specialist Surgical Sciences, Fondazione IRCCS Ca’ Granda Ospedale Maggiore Policlinico, 20122 Milan, Italy; ludovica.battilocchi@policlinico.mi.it (L.B.); annaco812@gmail.com (A.C.); lorenzo.gaini@unimi.it (L.M.G.); mirko.alde@unimi.it (M.A.); lorenzo.pignataro@unimi.it (L.P.); sara.torretta@unimi.it (S.T.); 3 Department of Clinical Sciences and Community Health, University of Milan, 20122 Milan, Italy

**Keywords:** type 2 inflammation, CRSwNP, nasal cytology, biologics, eosinophils

## Abstract

**Background/Objectives**: In recent years, the recognition that type 2 inflammation plays a leading role in CRSwNP has enabled the more tailored treatment of the disease through improved patient endotyping. We studied 45 patients with severe CRSwNP who were treated with dupilumab or mepolizumab. The aim was to evaluate the efficacy of these treatments on endoscopic, clinical and patient reported parameters, and to assess whether nasal cytology could be useful for identifying responsive patients and monitoring their response to biologic drugs. **Methods**: Follow-up visits were scheduled at baseline (T0), and at 3 (T3), 6 (T6), 12 (T12), and 24 months (T24). At each visit, patients underwent blood analysis, nasal endoscopy, and nasal scraping for cytology. They also completed the SNOT-22 questionnaire, a visual analog scale (VAS) for nasal obstruction and smell perception, and the Asthma Control Test (ACT) test in cases of concomitant asthma. **Results**: Biological therapy demonstrated broad efficacy in disease management, based on both clinical and cytological findings. The Nasal Polyp Score, SNOT-22 questionnaire, VAS scores for nasal obstruction and smell, and ACT score showed progressive improvement. Blood eosinophil counts and total IgE levels also decreased over time (T0 vs. T24: *p* = 0.008 and *p* < 0.001, respectively). At nasal cytology, a reduction in eosinophil cell count and in the mixed mast cell–eosinophil pattern during treatment with both biologics were observed (T0 vs. T24: *p* < 0.001). Positive effects were typically recorded within six months of treatment and were sustained after two years. **Conclusions**: Although the histological evaluation of infiltrated tissues remains the gold standard for assessing mucosal eosinophilia, nasal cytology appears to be a simpler, non-invasive, and repeatable method for evaluating local eosinophilia. Identifying endotypes and assessing the severity of inflammation are crucial for predicting the efficacy of different treatment options.

## 1. Introduction

Precision medicine is becoming the new standard of care, particularly with the availability of innovative therapies such as biologics for the treatment of chronic rhinosinusitis with nasal polyposis (CRSwNP). In recent years, the characterization of the immune response and underlying pathophysiological mechanisms in CRSwNP—known as “endotyping”—has enabled the identification of patient subgroups more likely to respond to specific treatments, thereby supporting a tailored clinical approach to the disease [1].

The current approach to CRSwNP proposes the differentiation between type 2 (eosinophilic) and non-type 2 inflammatory response. Elevated levels of tissue and peripheral blood eosinophils (polyp tissue eosinophils ≥ 10 per high-power field or blood eosinophils ≥ 150 cells/µL) and serum IgE (total IgE ≥ 100 IU/mL) are considered signs of type 2 inflammation, and patients should be considered for biologic therapy for the treatment of CRSwNP [1]. Although type 2 inflammation is present in approximately 80% of Caucasian patients with CRSwNP, about 20% may have disease driven by non–type 2 mechanisms, such as type 1 or type 3 inflammation [2]. A systematic review by Chong et al. [3] reported response rates of 50–70% in CRSwNP patients with comorbid asthma undergoing biologic therapy, indicating that not all patients benefit equally. Although the currently available biomarkers have limited utility in routine clinical practice and predicting an individual patient’s response to biologics is currently impossible, identifying CRSwNP with underlying type 2 inflammation is essential to ensure that only patients likely to benefit receive biologic treatment.

A comprehensive understanding of the specific interleukin and chemokine profiles, as well as the histopathological changes involved in the type 2 inflammatory cascade—responsible for the most severe cases of CRSwNP—is essential for identifying patients and treating them according to their disease endotype [4]. It is well established that the key cytokines in this inflammatory pathway are IL-4, IL-5, and IL-13. These cytokines contribute to the loss of cellular differentiation, reduced junctional integrity, and impaired innate immune defenses [5].

Currently, in Italy, two biologic drugs are approved for the treatment of CRSwNP associated with type 2 inflammation: dupilumab and mepolizumab [6]. Dupilumab is a monoclonal antibody targeting the IL-4 receptor alpha subunit, thereby inhibiting the actions of both IL-4 and IL-13. It is administered subcutaneously every two weeks at a dosage of 300 milligrams. Mepolizumab is a monoclonal antibody against IL-5, administered subcutaneously every four weeks at a dosage of 100 milligrams.

Histopathological studies have shown that 80–90% of CRSwNP patients exhibit prominent eosinophilia, both in the nasal polyp tissue [7] and in nasal smears [8,9]. The study of nasal immunoinflammation, which can be easily assessed through nasal cytology, therefore represents an important tool for improving endotyping and monitoring treatment response [10].

Several investigations have demonstrated a correlation between tissue eosinophilia and disease severity [11,12]. However, to date, there are limited data on the impact of monoclonal antibodies on nasal cytology and the inflammatory infiltrate in patients with CRSwNP. The aim of this study was (1) to assess the clinical impact in CRSwNP patients treated with the aforementioned biologics over a 24-month period and (2) to evaluate the cellular changes in the nasal inflammatory infiltrate through nasal cytology. Although this study does not compare the efficacy of the two biologics used, it reports for the first time the nasal cellular changes via cytology during therapy with these drugs for CRSwNP, monitoring their therapeutic effects over a 24-month follow-up.

## 2. Materials and Methods

### 2.1. Study Design

This is a prospective, monocentric observational study evaluating the clinical effects of biologic treatments—specifically dupilumab and mepolizumab—in patients with CRSwNP, with a particular focus on changes observed in nasal cytology. We enrolled patients attending the outpatient clinic for nasal polyposis at the Division of Otolaryngology, Fondazione Ca’ Granda Ospedale Maggiore Policlinico, Milan, Italy, who met the criteria for severe uncontrolled CRSwNP according to the guidelines of the Italian Medicines Agency (AIFA) for the prescription of biologics [6].

All participants signed an informed consent.

### 2.2. Inclusion Criteria

Subjects over 18 years of age with severe CRSwNP, defined by extensive bilateral nasal polyposis (Nasal Polyps Score [NPS] ≥ 5) and a significant impact on quality of life (Sino-Nasal Outcome Test-22 [SNOT-22] ≥ 50), who did not achieve adequate disease control with oral corticosteroids and/or surgery, or who experienced failure or intolerance to previous medical treatments (i.e., at least two cycles of systemic corticosteroids in the past year). Most patients were using intranasal corticosteroids (INCSs) and were adherent to the treatment.

### 2.3. Exclusion Criteria

Patients currently undergoing immunosuppressive therapy, those with chronic autoimmune diseases requiring long-term oral corticosteroid use, pregnant individuals, and those with allergic fungal rhinosinusitis.

### 2.4. Clinical Assessment

Patients were regularly referred and followed up at the “Type-2 Disease Centre”, a multidisciplinary study group within the hospital that manages individuals with type 2-related diseases [13].

General patient data were collected, including age, sex, smoking status, allergies (total and specific IgE to the main inhalant allergens were measured, in most cases), drug intolerances, and comorbidities—particularly the presence of other type 2 (Th2) inflammatory diseases such as asthma, atopic dermatitis, and eosinophilic esophagitis. Additional information, including non-steroidal anti-inflammatory drug–exacerbated respiratory disease (NSAID-ERD), history of nasal surgery, and current topical or systemic therapies, was also recorded.

Follow-up visits were scheduled at study inclusion (T0), and subsequently at 3 months (T3), 6 months (T6), 12 months (T12), and 24 months (T24).

At baseline and at each follow-up visit, patients underwent blood analysis and nasal endoscopy, and completed the Sino-Nasal Outcome Test-22 (SNOT-22) questionnaire and Visual Analog Scale (VAS) assessments for smell and nasal obstruction, as detailed below.

To achieve patient endotyping, the following blood tests were performed: absolute eosinophil count, lymphocyte subpopulations (T, B, and NK cells), liver enzymes, serum proteins, creatinine, C-reactive protein (CRP), immunoglobulins (IgA, IgG, IgM, IgE), peri-nuclear anti-neutrophil cytoplasmic antibodies (p-ANCA), and eosinophil cationic protein (ECP).

At baseline and during scheduled visits, fiberoptic nasal endoscopy was performed using a rhinolaryngoscope (Olympus ENF rhinolaryngo-fiberscope type GP; 33 cm in length, 3.4 mm in diameter; Olympus Medical Systems Corporation, Tokyo, Japan) to assess the Bilateral Endoscopic Nasal Polyp Score (NPS) according to EPOS/EUFOREA 2023 guidelines [14].

Briefly, the endoscopy scores were calculated by summing the unilateral scores of each nostril as follows:

- Score 0: no polyps;
- Score 1: small polyps in the middle meatus not extending below the inferior border of the middle turbinate;
- Score 2: polyps reaching below the lower border of the middle turbinate;
- Score 3: large polyps reaching the lower border of the inferior turbinate or polyps medial to the middle turbinate;
- Score 4: large polyps causing near-complete congestion or nasal obstruction of the inferior meatus.

The total NPS was the sum of the scores from both nasal fossae.

Additionally, nasal endoscopy included an assessment of the patency of surgical sinusotomies, as well as evaluation of inflammatory signs such as mucosal edema, crusting, and mucinous secretions [14].

All patients underwent a maxillofacial computed tomography (CT) scan, with the most recent scan performed within six months prior to enrollment. The CT scans were used to evaluate the extent of previous surgery—graded according to the Amsterdam Classification of Completeness of Endoscopic Sinus Surgery (ACCESS score) [15], the inflammatory involvement of the paranasal sinuses and ostiomeatal complex—graded using the Lund-Mackay classification [16], and to identify any possible iatrogenic sequelae.

Chronic rhinosinusitis (CRS) staging was determined using the Lund-Mackay system. Each sinus group is graded on a scale from 0 to 2: 0 indicating no abnormality, 1 indicating partial opacification, and 2 indicating total opacification. The ostiomeatal complex is scored as 0 if not obstructed or 2 if obstructed. The total score ranges from 0 to 24, with each side scored separately. A Lund-Mackay score of ≥4 was considered indicative of chronic rhinosinusitis [16].

The Sino-Nasal Outcome Test-22 (SNOT-22) is a quality of life questionnaire specifically designed for rhinosinusitis. It consists of 22 items, each scored from 0 to 5, with total scores ranging from 0 to 110 [17]. Lower scores indicate less impact on quality of life, while scores above 40 are associated with significant impairment. This questionnaire was completed by the patients at each visit.

The Visual Analog Scale (VAS) was used to assess the severity of main nasal symptoms and smell. Scores were categorized as mild impairment (0–3), moderate impairment (>3–7), and severe impairment (>7–10).

Nasal cytology, a non-invasive diagnostic tool, was performed at baseline (T0) and at follow-up visits (T3, T6, T12, and T24) to evaluate nasal inflammatory infiltration and identify the predominant cell types within the nasal inflammatory infiltrate.

Samples were obtained by bilaterally scraping the inferior turbinates using a disposable plastic nasal curette (Rhinoprobe^®^: Arlington Scientific, Springville, UT, USA). Nasal smears were immediately prepared by gently swiping the curette across glass slides, then air-dried and stained using the May-Grunwald–Giemsa Quick stain (Bio-Optica, Milan, Italy).

Observations were performed under oil immersion using an Olympus BH-2 optical microscope at a magnification of 1000× to identify eosinophils, mast cells, neutrophils, bacteria, spores, and other elements. For the rhinocytogram analysis, at least fifty fields were examined. The presence of each cell type was evaluated. The predominant cellular pattern was identified for each patient over time, allowing the identification of five cytologic phenotypes: (1) eosinophils; (2) eosinophils and mast cells; (3) neutrophils; (4) neutrophils and eosinophils; and (5) neutrophils and bacteria. A negative nasal cytology was also recorded. Particular attention was paid to eosinophils, whose count was expressed as the mean number observed in at least three of the richest high-power fields (1000× magnification), reported as the number of eosinophils per high-power field (hpf) [18]. At baseline, the Clinical Cytological Grading (CCG) was calculated for each patient [19]. This score is based on both nasal cytological findings and comorbidities—including asthma, allergy, and ASA (acetylsalicylic acid) sensitivity—with a global score of 1–3 indicating low grade, 4–6 moderate, and >7 severe disease [20].

Patients with concomitant asthma completed the Asthma Control Test (ACT), which scores range from 5 (poorly controlled asthma) to 25 (completely controlled asthma). Higher scores reflect better asthma control, with an ACT score ≥ 20 indicating well-controlled asthma [21].

The efficacy of treatment with biological drugs was evaluated according to EPOS/EUFOREA guidelines [14]: briefly, a response was considered good or excellent when, after six months of biologic treatment, there was an improvement in at least one symptom/score (NPS decrease ≥ 1, SNOT-22 reduction ≥ 8.9), and this improvement was judged satisfactory by the patient. The response was considered poor or moderate if no nasal symptom improvement was observed, or if the impact on sinonasal symptom/scores was less than the thresholds mentioned above.

### 2.5. Criteria for Selecting Biologics

Dupilumab was the first choice in cases of severe nasal polyposis with concomitant allergic asthma, elevated IgE levels or allergic sensitization, NSAID-ERD, or early relapse after surgery.

Mepolizumab was preferred in cases of severe nasal polyposis with significant blood eosinophilia (≥250–300/µL), presence of severe eosinophilic asthma as a comorbidity -independent by allergy/IgE status-, and when the endotype was clearly eosinophil-driven.

The initial choice of biologic was also based on comorbidities and, therefore, on the dominant disease and symptoms. The treatment options were presented and discussed with the patient, also taking into account his preferences.

### 2.6. Statistical Analysis

Demographic and clinical characteristics were summarized as means with standard deviations (SDs) for normally distributed continuous variables, or as absolute frequencies and percentages for categorical variables. Differences in percentages were analyzed using Fisher’s exact test, while differences in means over time within the same subjects were assessed using repeated-measures ANOVA. Correlation analyses were performed using Pearson’s correlation coefficient. Statistical significance was set at *p* < 0.05. All analyses were conducted using R software (version R.4.5.1).

## 3. Results

The study included 45 patients (mean age: 58.6 ± 13.2 years; range: 19–80) affected by severe CRSwNP and eligible for treatment with biological drugs. Of these, 21 were female and 24 were male.

Most patients (84.4%) had undergone previous surgeries for nasal polyposis, with a mean of 2.1 ± 0.9 surgeries per subject. Approximately 70% of patients used systemic corticosteroids in the past year (≥2 courses, mean treatment duration 7–10 days). Thirty-two subjects (73.8%) suffered from asthma, and 19 (42.2%) from allergies; sensitization to dust mites was observed in 57.9% of patients, and to grass pollen in 52.6%. Among patients with concomitant asthma, the mean ACT score at admission was 20.4 ± 4.9. Baseline demographics and clinical characteristics are shown in Table 1.

For the treatment of severe CRSwNP, 23 patients received dupilumab and 22 received mepolizumab.

**Figure 1:**
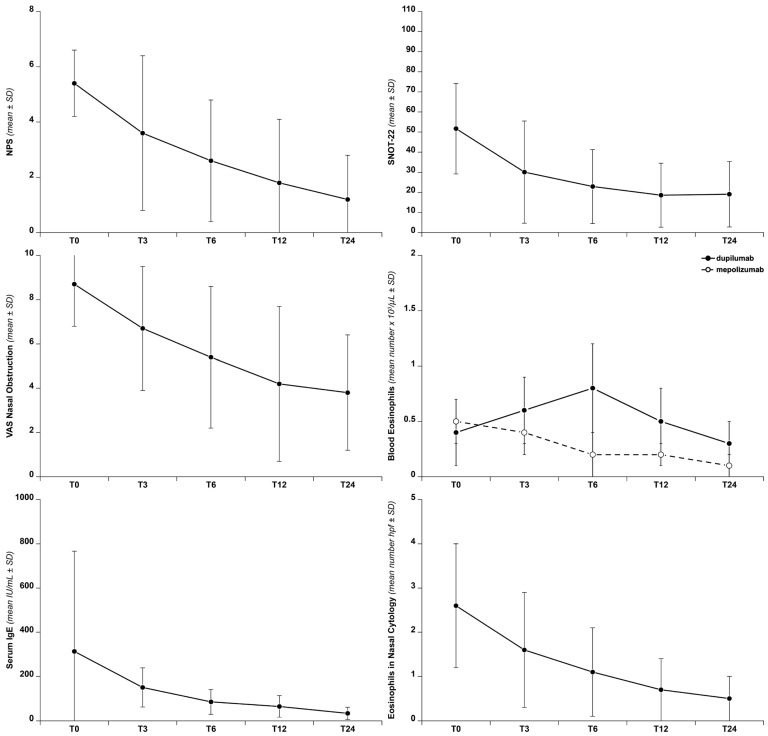
Outcomes over time of NPS, SNOT-22, VAS for nasal obstruction, blood eosinophils, serum IgE, and eosinophils in nasal cytology.

Figure 1 shows the progression of the main clinical parameters evaluated over time.

Biological therapy has demonstrated broad efficacy in disease management, as evidenced by both clinical and cytological findings. In particular, treatment with biologic drugs led to a significant and gradual improvement in the Nasal Polyp Score (NPS), which decreased (T0 vs. T3: *p* < 0.001, T0 vs. T6: *p* = 0.06, T6 vs. T12: *p* = 0.09, T12 vs. T24: *p* = 0.15; and T0 vs. T24: *p* < 0.001).

Similarly, SNOT-22 scores showed a significant reduction, (T0 vs. T3: *p* < 0.001, T3 vs. T6: *p* = 0.13, T6 vs. T12: *p* = 0.24, T12 vs. T24: *p* = 0.88 and T0 vs. T24: *p* < 0.001), indicating that the improvement in SNOT-22 scores was sustained throughout the study period.

The VAS for nasal obstruction also decreased over time (T0 vs. T3: *p* < 0.001, T3 vs. T6: *p* = 0.04, T6 vs. T12: *p* = 0.09, T12 vs. T24: *p* = 0.54 and T0 vs. T24: *p* < 0.001), indicating improved nasal patency following biologic therapy. Correlation analysis showed a significant relationship between NPS and VAS for nasal obstruction score (*p* = 0.05) (Table 2).

In asthmatic patients, ACT scores also showed a progressive improvement over time (T0 vs. T3: *p* = 0.14, T3 vs. T6: *p* = 0.61, T6 vs. T12: *p* = 0.09, T12 vs. T24: *p* = 0.54 and T0 vs. T24: *p* < 0.001).

A significant correlation was found between the SNOT-22 and ACT scores (*p* = 0.01).

At T0, 38 patients (84.4%) had serum eosinophil counts > 150 cells/mm^3^. Following treatment with biologic agents, eosinophil levels showed a long-term decrease overall. However, in patients treated with dupilumab, a transient increase in mean eosinophil counts was observed, particularly at T3 and T6 (T0 vs. T3: *p* = 0.008, T3 vs. T6: *p* = 0.01, T6 vs. T12: *p* = 0.01, T12 vs. T24: *p* = 0.005 and T0 vs. T24: *p* = 0.005). In contrast, patients treated with mepolizumab exhibited a progressive decrease in eosinophil levels over time T0 vs. T3: *p* = 0.004, T3 vs. T6: *p* = 0.004, T6 vs. T12: *p* = 0.002, T12 vs. T24: *p* < 0.001 and T0 vs. T24: *p* = 0.002.

No significant correlation was found between blood eosinophil count and symptom severity as measured by the SNOT-22 score (*p* = 0.18). Additionally, baseline eosinophil levels did not significantly differ between patients with CRSwNP and asthma and those with CRSwNP alone (*p* = 0.76).

IgE levels showed a statistically significant decline over time (T0 vs. T3: *p* = 0.02, T3 vs. T6: *p* < 0.001, T6 vs. T12: *p* = 0.06, T12 vs. T24: *p* < 0.001 and T0 vs. T24: *p* < 0.001).

Regarding nasal cytology, at baseline, 36 patients (80%) presented with eosinophilia (defined as >1 eosinophil/high power field [hpf], based on the mean count across the three richest fields). Over the course of treatment with both biologics, there was a notable reduction in eosinophil counts within the nasal cellular composition compared to baseline. Additionally, a similar reduction was observed in the mast cell–eosinophilic cytological pattern.

The mean number of eosinophils per high-power field (hpf) decreased (T0 vs. T3: *p* < 0.001, T3 vs. T6: *p* = 0.04, T6 vs. T12: *p* = 0.03, T12 vs. T24: *p* = 0.14 and T0 vs. T24: *p* < 0.001). The reduction in eosinophils observed in nasal cytology was consistent with improvements in both the SNOT-22 score and the Nasal Polyp Score (NPS) in treated patients. The progressive decrease in eosinophils and in the mixed mast cell/eosinophil pattern on nasal cytology was mainly observed in patients with a good or excellent response to treatment, whereas patients with a poor or moderate response predominantly showed neutrophils at baseline (8 out of 9, i.e., 88.9%). Table 3 provides an overview of the trends in nasal cytology cell subtypes over time in CRSwNP patients treated with biologics.

**Figure 2:**
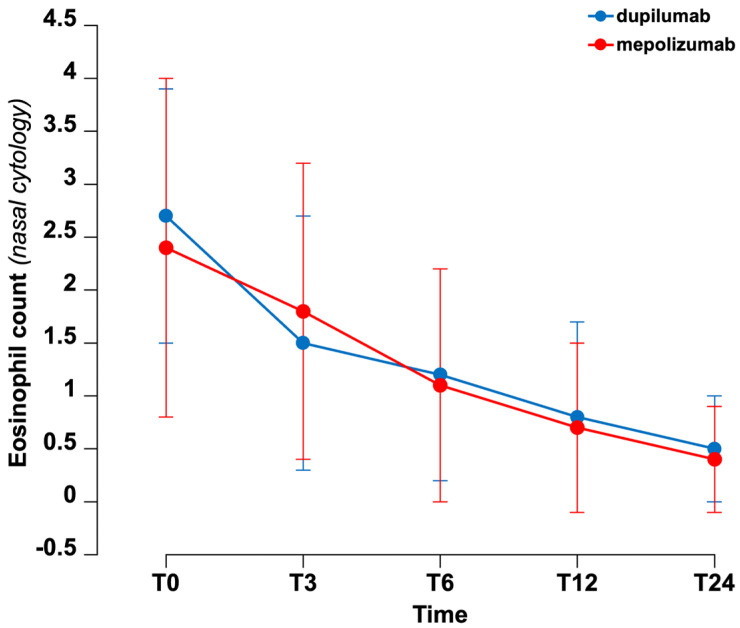
Comparison of eosinophil counts in nasal cytology at different time points (mean count across at least three richest fields).

When comparing the decrease in eosinophils observed via nasal cytology between patients treated with dupilumab and those treated with mepolizumab, no statistically significant differences were found (Figure 2).

After one year of biologic therapy, 9 out of 45 patients (20%) showed complete negative nasal cytology. This number increased to 12 patients (26.7%) after two years of treatment.

**Figure 3:**
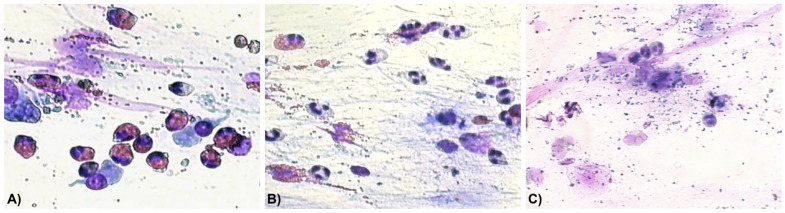
Examples of nasal cytology: (**A**) the inflammatory picture is dominated by eosinophils; (**B**) a mixed inflammation with neutrophils and eosinophils; (**C**) neutrophils and associated bacteria. Magnification: 400×.

Neutrophil counts increased during treatment with both biologics, and some patients also presented with bacteria and neutrophils in nasal cytology samples, whereas no bacterial presence was detected at T0 (Figure 3).

When evaluating the correlation between local eosinophilia (nasal cytology) and systemic eosinophilia (blood eosinophil count), no significant association was found (*p* = 0.89). Local eosinophilia was significantly related to the CCG score (*p* = 0.02).

Overall, the response to treatment was rated as good or excellent in 36 patients (80%), and poor or moderate in 9 (20%). In two patients treated with dupilumab, therapy was discontinued due to the onset of eosinophilic granulomatosis with polyangiitis (EGPA). Similarly, EGPA developed in two patients treated with mepolizumab; in these cases, the biologic dosage was increased to 300 mg every 4 weeks. Three patients who developed EGPA were asymptomatic, while one developed myocarditis during treatment with dupilumab.

A transient increase in blood eosinophils was observed in 11 patients (24.4%) receiving dupilumab, which resolved in subsequent months without requiring therapy discontinuation. Due to persistent eosinophilia, four patients were switched from dupilumab to mepolizumab, while one patient switched from mepolizumab to dupilumab due to a poor response.

## 4. Discussion

CRSwNP is highly heterogeneous and comprises distinct disease subtypes. Nevertheless, type 2 inflammation is present in most patients [1,2]. Eosinophilic inflammation in the nasal mucosa of CRSwNP patients has been associated with greater symptom severity, poorer disease control, and a reduced response to both medical and surgical treatments [18]. Blood and tissue eosinophilia play a central role in sustaining type 2 inflammation, and biologics such as dupilumab and mepolizumab primarily work by reducing eosinophilic inflammation.

In Italy, patients with CRSwNP can access biologic treatments in cases of severe (Nasal Polyp Score ≥ 5 or SNOT-22 score ≥ 50) and uncontrolled disease—such as in patients who have not achieved disease control with oral corticosteroids (OCSs) and/or surgery [6,22]. In our case series, the most appropriate biologic treatment for each patient is selected according to the EPOS/EUFOREA guideline criteria [14].

To date, only a few studies on the effects of biologics in CRSwNP have included nasal cytology as a simple method to evaluate changes in the patients’ nasal inflammatory infiltrate, with most focusing on dupilumab treatment.

Overall, these studies found improvements in both subjective and objective parameters after biological treatment and reported a reduction in local nasal inflammation, as measured by eosinophil and neutrophil counts [22,23,24,25]. During treatment with both biologics, we observed a notable reduction in eosinophil counts, along with a similar decrease in the mast cell–eosinophilic cytological pattern within nasal inflammatory cells compared to baseline. These findings are consistent with previous reports; moreover, in line with those studies, we did not find any correlation between eosinophil counts from nasal cytology and blood eosinophilia.

Even fewer studies have been conducted using biologics other than dupilumab and including nasal cytology. Mepolizumab and benralizumab, in addition to improving both sinonasal and asthma symptoms, have also been shown to induce a significant reduction in nasal eosinophil counts [26,27].

Consistent with the investigations cited above, our study demonstrates that both dupilumab and mepolizumab significantly improve clinical and patient-reported outcomes. Positive effects are typically achieved within six months of treatment and are sustained over a two-year period. Probably due to the small sample size, we were unable to detect differences in treatment response between dupilumab and mepolizumab. Our research also focused on the impact of dupilumab and mepolizumab on eosinophils and other inflammatory cells as assessed by nasal cytology: among patients demonstrating a good or excellent response to treatment, a progressive decrease in eosinophils and in the mixed mast cell/eosinophil pattern on nasal cytology was observed. This suggests tissue remodeling induced by the suppression of type 2 inflammation.

At baseline, only a minority of patients (20%) showed neutrophils; these patients were predominantly those who later exhibited a poor or moderate response to treatment. However, it is known that a subset of CRSwNP patients who are difficult to treat are characterized by tissue neutrophilic inflammation and elevated levels of interleukin-8 (IL-8); thus, the presence of neutrophils in nasal cytology may be associated with disease refractoriness [28,29].

The absence of eosinophils on nasal cytology prior to initiating treatment may serve as a predictor of a poor or moderate response to biologics that act by targeting type 2 inflammation. Although these are small numbers, the observation is intriguing and warrants further investigation in larger studies. However, it must also be considered that several patients were using INCS at the time of inclusion in the study, which may have influenced eosinophil counts observed in nasal cytology.

During the course of biologic therapy, neutrophils began to appear in nasal cytology, indicating a shift in the inflammatory cell profile. This rise in neutrophils is likely attributable to the imbalance between type 2 and non-type 2 inflammation induced by the biologic drugs. Similar results have been reported by Pecorari G. et al. [30], who demonstrated an increase in neutrophils on nasal cytology after 12 months of dupilumab treatment for CRSwNP, independently of regular intranasal corticosteroid use.

In some patients, both neutrophils and associated bacteria were observed after treatment. This may be explained by the reduction in eosinophils, which play a role in maintaining immune homeostasis and are also involved in suppressing bacterial and viral infections [31].

After two years of treatment, negative nasal cytology was achieved in 26.7% of patients.

The main limitation of our study is the relatively small sample size, which is inherent to its observational and monocentric design, as well the lack of a whole comparative analysis of clinical outcomes between the two biologics drugs used. Nevertheless, the primary aim of the study was not to compare the efficacy of the two biological agents (already addressed in clinical trials and meta-analyses on larger cohorts), but rather to highlight the use of nasal cytology as a simple, non-invasive method to better characterize patients and potentially predict the response to biological therapy. Another limitation of the study is the use of INCS by most patients, which may have influenced the results of nasal cytology, as already mentioned above.

Although histological evaluation of infiltrated tissue remains the gold standard for assessing mucosal eosinophilia, its invasiveness limits its routine use in clinical practice. Nasal cytology, by contrast, appears to be a simpler, non-invasive, and repeatable method for evaluating local eosinophilia. The analysis of sinonasal secretions has gained growing interest in recent years, as these are easy to obtain compared with tissue sampling [32].

The assessment of the degree of eosinophilia through nasal cytology is still not widespread among rhinologists, though since several reports have confirmed a correlation between tissue eosinophilia and cytological eosinophil counts, the study of inflammatory cells via nasal cytology may represent a useful surrogate to detect local eosinophilia and to monitor the response to biologic treatment.

## 5. Conclusions

Although studies investigating the impact of biologics on nasal cytology are still limited, our findings support the potential role of nasal cytology as a useful, practical tool for monitoring patients undergoing biologic treatment for CRSwNP. The otolaryngologists can investigate nasal secretions for eosinophilia as a guide to the presence of type 2 inflammation. Further research is warranted to confirm and expand upon these results.

## Figures and Tables

**Table 1:** Patients’ baseline demographics and clinical characteristics are reported as numbers and percentages for categorical variables, and as mean ± standard deviation (SD) for continuous variables.

	All (45)	Dupilumab (23)	Mepolizumab (22)
Female	21 (46.6)	13 (56.5)	8 (36.4)
Age (years)	58.6 ± 13.2	58.9 ± 13.6	58.3 ± 13.1
Family history of allergies	19 (42.2)	6 (26.1)	13 (59.1)
NSAID intolerance	17 (37.8)	9 (39.1)	8 (36.4)
Asthma	32 (71.1)	17 (73.9)	15 (68.2)
ACT (0–25)	20.4 ± 4.9	20.4 ± 4.8	20.3 ± 5.1
Previous sinonasal surgeries	2.1 ± 0.9	2.2 ± 1.1	1.9 ± 0.8
Bilateral NPS (0–8)	5.4 ± 1.2	5.7 ± 1.2	5.2 ± 1.3
Lund-McKay score (0–24)	12.6 ± 5.8	13.4 ± 6.2	11.8 ± 5.6
Clinical Cytological Grading	4.6 ± 1.2	4.7 ± 1	4.5 ± 1.3
SNOT-22 (0–110)	51.7 ± 22.5	57.3 ± 17	46.1 ± 6.2
VAS nasal obstruction (0–10)	8.7 ± 1.9	9 ± 1.9	8.3 ± 2.3
VAS smell (0–10)	7.6 ± 2.5	7.8 ± 2.2	7.4 ± 2.8
Blood eosinophils (×10^3^/µL)	0.4 ± 0.2	0.4 ± 0.3	0.4 ± 0.2
IgE level (IU/mL)	313.2 ± 453.5	201.4 ± 370.4	425.2 ± 519.7

**Table 2:** Pearson’s correlation coefficients (r) and *p*-values for the main parameters used in evaluating treatment efficacy.

Variables	r	*p*-Value
Blood eosinophils vs. nasal eosinophils	0.03	0.89
Blood eosinophils vs. SNOT-22	−0.28	0.18
Blood eosinophils vs. NPS	−0.19	0.32
Nasal eosinophils vs. SNOT-22	−0.03	0.89
Nasal eosinophils vs. NPS	0.18	0.31
Nasal eosinophils vs. VAS nasal obstruction	−0.12	0.53
Nasal eosinophils vs. CCG	0.35 *	0.02
NPS vs. SNOT-22	0.21	0.32
NPS vs. VAS nasal obstruction	0.38 *	0.05
NPS vs. CCG	0.05	0.76
SNOT-22 vs. VAS nasal obstruction	0.18	0.43
SNOT-22 vs. CCG	0.05	0.80
SNOT-22 vs. ACT	−0.52 *	0.01

* Statistically significant correlation (*p* < 0.05).

**Table 3:** Phenotypes identified at nasal cytology over the treatment course with biological drugs (number of patients, percentages).

	T0	T3	T6	T12	**T24**
Eosinophils	11 (24.4)	8 (17.7)	5 (11.1)	1 (2.2)	0 (0)
Eosinophils + mast cells	7 (15.6)	5 (11.1)	2 (4.4)	0 (0)	0 (0)
Neutrophils	9 (20)	15 (33.3)	16 (35.6)	20 (44.4)	22 (48.9)
Neutrophils + eosinophils	18 (40)	14 (31.1)	11 (24.4)	6 (13.3)	0 (0)
Neutrophils + bacteria	0 (0)	2 (4.4)	6 (13.3)	9 (20)	11 (24.4)
Negative nasal cytology	0 (0)	1 (2.2)	5 (11.1)	9 (20)	12 (26.7)

## Data Availability

The original contributions presented in the study are included in the article; further inquiries can be directed to the corresponding author.

## Abbreviations

The following abbreviations are used in this manuscript:

CRSwNP	Chronic rhinosinusitis with nasal polyposis
OCS	Oral corticosteroids
INCS	Intranasal corticosteroids
NPS	Nasal Polyp Score
VAS	Visual Analogue Scale
SNOT-22	Sino-Nasal Outcome Test-22
ACT	Asthma Control Test
CCG	Clinical Cytological Grading
